# Tongue image analysis for early warning of neonatal necrotizing enterocolitis: a translational perspective integrating tongue phenotyping and microbiome dynamics

**DOI:** 10.3389/fped.2026.1982364

**Published:** 2026-10-29

**Authors:** Jie Yang, Jing Li, Yuanyuan Lv, Linqi Cai, Wei Gao

**Affiliations:** 1 Department of Preventive Medicine, College of Integrative Chinese and Western Medicine, Anhui University of Chinese Medicine, Hefei, China; 2 Department of Respiratory Medicine, The First Affiliated Hospital of Anhui University of Chinese Medicine, Hefei, China; 3 Department of Pediatric Surgery, Anhui Provincial Children’s Hospital, Hefei, China

**Keywords:** early warning, microbiome, necrotizing enterocolitis, quantitative tongue image analysis, tongue phenotype

## Abstract

Necrotizing enterocolitis (NEC) remains a life-threatening challenge in neonatology, primarily due to the lack of sensitive early biomarkers that precede overt clinical and radiological signs. This Perspective proposes a hypothesis-generating, non-invasive early-warning framework by integrating quantitative tongue image analysis with modern pathophysiological insights. We hypothesize a dynamic association between tongue phenotypic changes (e.g., a transition from pale to purplish color) and the progression of dysbiosis and intestinal microcirculatory disturbance, which correspond to two distinct but potentially complementary pathways. The proposed biological link rests on evidence for the candidate oral–gut microbial relationship, with such evidence derived primarily from adult and non-neonatal studies; gut dysbiosis—known to precede NEC—may be associated with changes in the tongue-coating microbiome, offering a potential remote yet accessible window into intestinal health. We outline a three-phase translational research roadmap: (I) prospective discovery of tongue-image biomarkers and establishment of dynamic baselines for each infant, (II) development of an exploratory machine-learning-based risk prediction model, and (III) mechanistic exploration using multi-omics approaches and animal models. Critically, we propose three testable hypotheses regarding temporal changes in tongue colorimetric and microbial parameters before NEC onset. If prospectively validated, this approach may eventually contribute to a dynamic bedside monitoring tool for NEC risk stratification.

## 1 Introduction

Necrotizing enterocolitis (NEC) is a critical clinical disorder in neonates, especially preterm infants, and remains a leading cause of gastrointestinal mortality in the neonatal intensive care unit (NICU) (1). Pathologically, NEC is characterized by intestinal mucosal necrosis and pronounced inflammatory activation, and its rapid progression threatens both short-term survival and long-term neurodevelopmental outcomes. Its pathogenesis is multifactorial, involving prematurity, dysregulated inflammatory responses, intestinal ischemia–hypoxia, microbial infection, and gut dysbiosis (2–4). Early diagnosis and prompt intervention are critical to improving clinical outcomes, yet conventional diagnostic tools often lack sensitivity in the early stages, resulting in delayed treatment. Therefore, developing a non-invasive, sensitive, and reliable early-warning system is an urgent unmet clinical need.

Tongue inspection has historically been used as a diagnostic approach and offers practical advantages for early disease detection owing to its simplicity and non-invasiveness. Modern research suggests a possible association between the tongue-coating microbiome and gut microbiota (5–7), whereby alterations in the tongue coating may mirror shifts in the structure and composition of gut microbiota, lending partial support to the candidate oral–gut microbial relationship.

Conventional visual tongue diagnosis suffers from limited reproducibility. By contrast, quantitative tongue analysis integrates digital image acquisition, image processing, and machine learning to objectively extract colorimetric, textural, and morphological features (8). This approach may aid in identifying candidate phenotypic signatures. However, its capacity to distinguish NEC from NEC-mimicking neonatal conditions (e.g., sepsis, feeding intolerance, and other intestinal disorders) remains to be established.

To our knowledge, no published studies have directly evaluated the association between tongue manifestations and neonatal NEC. To address the critical knowledge gap, this Perspective systematically explores quantitative tongue-image features as candidate digital biomarkers for early warning of NEC. Specifically, three key contributions are presented: (1) a hypothesis-generating rationale is proposed for tongue imaging as a potential early NEC monitoring tool via the candidate oral–gut microbial relationship; (2) a three-phase translational roadmap featuring a dynamic baseline design tailored for preterm infants is outlined; and (3) three quantifiable, falsifiable hypotheses are formulated to guide prospective cohort studies. This Perspective aims to foster interdisciplinary research at the intersection of neonatal medicine, biomedical engineering, and diagnostic innovation.

## 2 NEC: pathogenesis and unmet diagnostic needs

### 2.1 Multifactorial pathogenesis

NEC is a multifactorial syndrome, and prematurity remains its principal risk factor, with incidence inversely correlated with gestational age (9). Preterm neonates commonly exhibit underdeveloped intestinal barrier function, reduced gastric acid secretion, impaired gastrointestinal motility, and limited nutrient absorptive capacity. Diminished digestive enzyme activity and underdeveloped immune defenses render these infants particularly susceptible to mucosal injury.

In the immature intestine, microbial dysbiosis and exaggerated innate immune signaling interact with epithelial barrier dysfunction and impaired microvascular regulation, producing inflammation, tissue hypoxia, and progressive intestinal injury. Intestinal ischemia–reperfusion injury may contribute to the pathogenesis of NEC. In some high-risk infants, clinical conditions such as perinatal asphyxia, severe cardiopulmonary dysfunction, and shock may precipitate systemic hypoxia–ischemia, potentially leading to mesenteric vasoconstriction and reduced intestinal perfusion. Reperfusion may trigger an excessive burst of reactive oxygen species, exacerbating mucosal damage (10). Infection also plays a contributing role, as pathogenic microorganisms and their toxins can directly injure the intestinal epithelium and stimulate immune cells to release proinflammatory cytokines (11). Disruption of the gut microenvironment, commonly associated with delayed initiation of enteral feeding or prolonged exposure to broad-spectrum antibiotics, compromises the development of a balanced intestinal microbiota and predisposes to pathogenic overgrowth (12). Furthermore, inappropriate feeding practices, such as hyperosmolar formulas or rapid advancement of enteral feeds, impose significant stress on the immature gut (13, 14).

Beyond ischemia, inflammation, and dysbiosis, accumulating evidence implicates epithelial redox imbalance and stress-response pathways in NEC pathogenesis. Specifically, S1P-mediated regulation of both mitochondrial redox homeostasis and gut microbiota, elevated DUOX2 expression in NEC, and CHI3L1-mediated excessive autophagy in intestinal epithelial cells offer further mechanistic insights into this disease process (15–17).

### 2.2 Limitations of current diagnostic modalities

The initial presentation of NEC is often nonspecific, including feeding intolerance, gastric residuals, and lethargy. With disease progression, abdominal distension and hematochezia appear. Laboratory findings (thrombocytopenia, elevated C-reactive protein, procalcitonin, fecal calprotectin) and abdominal radiography (pneumatosis intestinalis, portal venous gas) remain diagnostic mainstays. Despite high specificity, the suboptimal sensitivity of abdominal x-rays restricts early detection (18). Ultrasonography offers superior sensitivity for portal venous gas and bowel wall assessment but remains operator-dependent and is not continuously available.

Contemporary biomarker research for NEC has focused on intestinal fatty acid-binding protein (I-FABP), fecal calprotectin, and a range of inflammatory mediators. Nevertheless, evidence supporting consistent diagnostic sensitivity and specificity remains limited, and multicenter validation of these markers for presymptomatic early warning is still lacking (19). Recent multi-omic investigations report serum ANXA2 as a promising exploratory biomarker, but its diagnostic performance requires further prospective multicenter confirmation (20).

## 3 Biological rationale for tongue-based NEC monitoring

### 3.1 Conceptual framework of intestinal-tongue relationship

Traditional Chinese medicine has historically used tongue inspection as a holistic diagnostic approach; however, this Perspective focuses on testable biomedical hypotheses. The tongue represents an accessible, microbially colonized mucosal surface whose phenotype may be influenced by systemic physiology and early-life microbial exposures. These properties underpin our hypothesis that tongue features may serve as a non-invasive window for identifying preclinical functional alterations relevant to NEC.

### 3.2 Candidate oral–gut microbial relationship and microcirculatory pathways

Two distinct but complementary pathways may link intestinal injury to tongue phenotypic changes in NEC: (i) a microbial/ecological pathway, whereby gut dysbiosis reshapes the tongue-coating microbiome and coating appearance; and (ii) a microcirculatory/systemic pathway, whereby intestinal hypoperfusion alters tongue blood perfusion and color. Evidence for these two pathways differs substantially and is discussed separately below.

#### 3.2.1 Microbial/ecological pathway

Associations between tongue-coating microbiota composition and gut microbial profiles have been reported, predominantly in adult and non-neonatal populations (21–23). We therefore refer to this as a candidate oral–gut microbial relationship, rather than a validated biological linkage. Gut dysbiosis, characterized by reduced *Bifidobacterium* and expansion of pathogenic taxa, is a recognized feature of NEC. Prior data show reduced gut microbial diversity in infants who later develop NEC, with microbial perturbations detectable before symptom onset (24, 25). Importantly, such microbial signatures are heterogeneous: pre-NEC low diversity is prominent in late-onset NEC, whereas consistent preclinical microbial markers are absent for early-onset disease (26).

In preterm infants, recent longitudinal studies show that oral microbial communities evolve rapidly and are influenced by gestational age, birth weight, feeding, delivery mode, and antibiotic exposure (27). Paired oral-intestinal sampling further reveals that oral and gut microbial communities follow separate developmental trajectories and occupy distinct ecological niches, supporting our cautious framing of this oral–gut linkage (28). Findings from cerebral ischemia–reperfusion injury models indicate that ischemic insult can trigger tongue-coating microbiota dysbiosis (29), raising the possibility that NEC-related intestinal ischemia–hypoxia may similarly remodel tongue microbial communities through systemic circulatory or inflammatory signaling. Preclinical multi-organ analyses suggest that gut microbial metabolites can drive systemic redox changes across distant organs (30), providing a conceptual framework for how intestinal dysbiosis might influence remote tissues through circulating mediators. However, this cross-organ signaling paradigm has not been evaluated in NEC, and it does not establish a tongue–gut axis.

#### 3.2.2 Microvascular/systemic pathway

Tongue color and surface microcirculation may reflect systemic perfusion status. In adults with septic shock, tongue ischemic changes correlate with enterocyte injury and adverse outcomes (31). These observations support the broader plausibility that tongue phenotypes may respond to systemic circulatory disturbances; however, they do not establish the tongue as a specific surrogate for intestinal microvascular perfusion. In NEC, intestinal ischemia–reperfusion and systemic hypoperfusion may perturb widespread mucosal microcirculation. As a highly vascularized mucosal tissue, the tongue may mirror these perfusion abnormalities via quantifiable color and microcirculatory signals, offering a potential means to identify subclinical intestinal ischemia.

Critically, tongue color changes are not NEC-specific. Confounding conditions include systemic hypoxemia, anemia, shock, vasopressor administration, sepsis, congenital heart disease, blood transfusion, worsening respiratory status, hemoglobin shifts, and peripheral hypoperfusion. These factors will be adjusted for in subsequent analyses. The central translational question is whether tongue phenotypes contain NEC-specific information beyond generalized cardiorespiratory or microcirculatory deterioration.

### 3.3 Positioning of tongue-image analysis relative to existing NEC surveillance

Quantitative tongue-image analysis is not intended to replace established NEC surveillance tools, but rather serves as a complementary, non-invasive bedside monitoring modality. Existing non-invasive modalities include splanchnic near-infrared spectroscopy (NIRS), bowel ultrasound, circulating biomarkers (e.g., C-reactive protein, procalcitonin, fecal calprotectin), and physiological monitoring. However, these approaches have important limitations: NIRS may be affected by abdominal-wall thickness and signal interference; bowel ultrasound is operator-dependent and not continuously available; circulating biomarkers often lack early sensitivity and intestinal specificity; and physiological monitoring is non-specific for intestinal injury. Tongue-image analysis may offer complementary advantages by enabling repeated bedside assessment of mucosal perfusion and microbial phenotypes, potentially improving temporal resolution and capturing early signals that precede overt clinical signs. Its incremental value relative to existing modalities will require prospective comparative validation.

## 4 Quantitative tongue phenotyping: technology and neonatal-specific challenges

To test these hypotheses, robust quantitative tools are required. The section reviews the technological foundation for tongue phenotyping and highlights the unique challenges posed by the neonatal population.

### 4.1 Standardized image acquisition for preterm infants

Quantitative tongue analysis relies on standardized image acquisition. Available devices range from dedicated tongue imagers (with controlled illumination and color calibration) to digital cameras and smartphones. Standardized protocols must control pre-acquisition factors (e.g., oral cleaning), patient posture, and lighting conditions to ensure data comparability. Protocols will adopt standardized color calibration (e.g., CIE L*a*b*), fixed imaging distance, and standardized timing for image capture relative to feeding.

Image acquisition will not require active tongue protrusion or routine oral manipulation. Whenever possible, images will be obtained with minimal handling by gently opening the mouth during routine nursing care. For extremely premature infants on respiratory support, daily acquisition may not always be feasible; sampling frequency will be adapted to clinical stability, with feasibility assessed during Phase I.

A key methodological consideration is that oral cleaning can modify both the appearance of the tongue coating and its microbiota. Therefore, two acquisition strategies will be predefined: native phenotype acquisition (imaging and microbiome sampling without pre-imaging cleaning) and standardized post-cleaning imaging. If cleaning is performed, it will be documented, and native versus post-cleaning datasets will be analyzed separately.

Existing tongue-image datasets and reference values are derived almost exclusively from adults; neonatal-specific acquisition workflows and population-matched reference datasets remain essential prerequisites for clinical translation.

### 4.2 Feature extraction and machine learning

The standard analytical workflow includes three core steps: image preprocessing, tongue-body segmentation, and quantitative feature extraction. Advanced image-processing techniques (grayscale adjustment, noise reduction, color correction) and segmentation algorithms enable precise delineation of the tongue body from the oral background. Feature extraction then quantifies three core domains of the tongue: color, texture, and morphology, providing objective, reproducible metrics that minimize inter-observer variability in visual assessment (32).

Building on these quantitative features, machine learning and deep learning algorithms may enable automated pattern recognition and dynamic risk stratification, potentially supporting longitudinal monitoring of disease progression. While these technologies are well established in adult populations, their adaptation to neonates requires overcoming unique challenges related to motion artifacts, the small oral cavity, and age-specific physiological reference ranges (33, 34).

## 5 Three-phase translational research roadmap

Given remaining uncertainties surrounding the hypothesized candidate oral–gut microbial relationship in NEC, we outline a structured three-phase methodological framework to systematically evaluate the utility of tongue image analysis for early warning of NEC and guide its clinical translation. Figure 1 illustrates the proposed mechanistic framework linking NEC biology to tongue phenotypic alterations.

**Figure 1:**
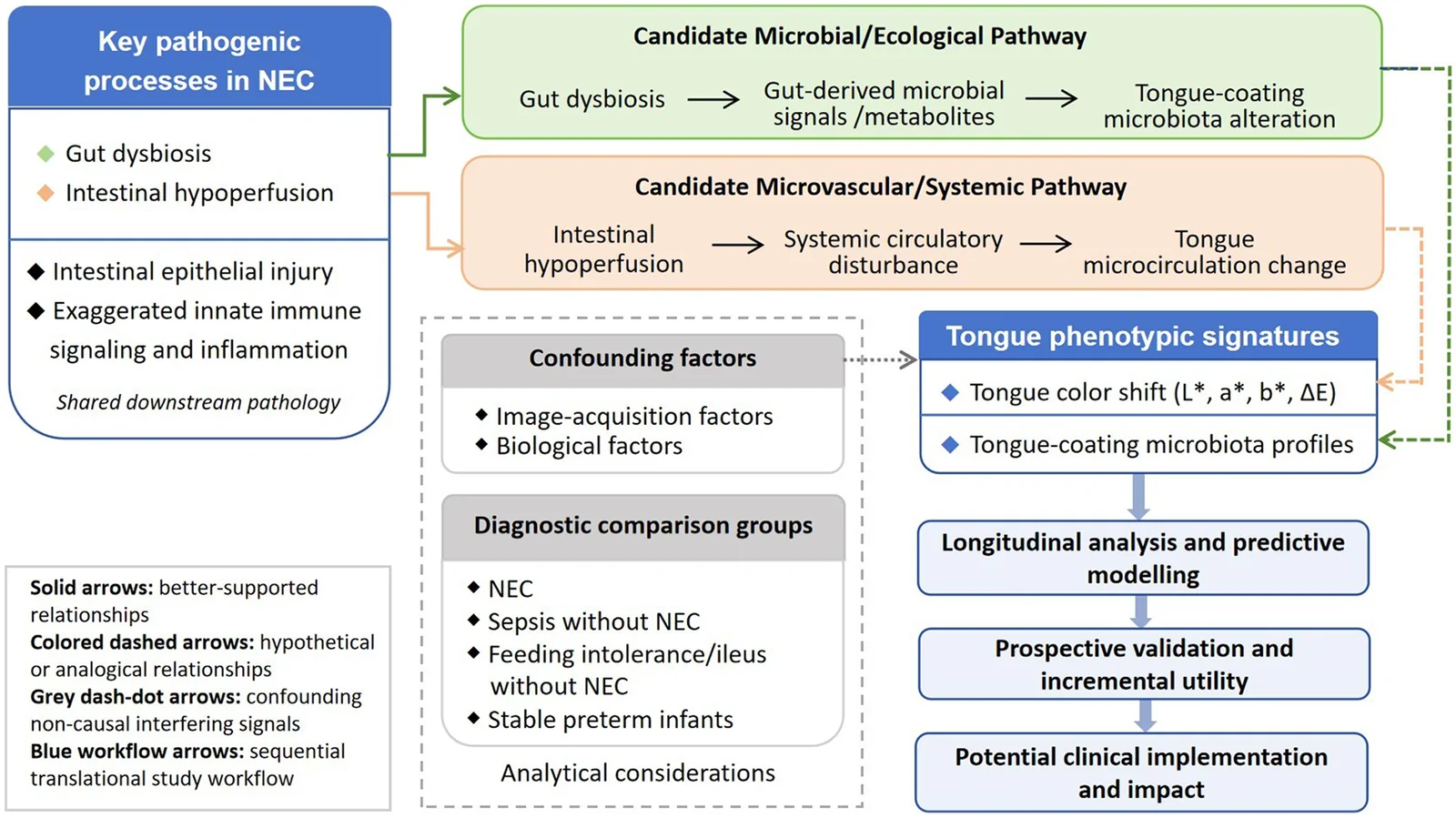
Mechanistic framework linking NEC biology to tongue phenotypic alterations via candidate microbial/ecological and microvascular/systemic pathways. Key pathogenic processes in NEC (left panel) are categorized into initiating drivers (gut dysbiosis and intestinal hypoperfusion) and shared downstream pathology (intestinal epithelial injury and exaggerated innate immune signaling/inflammation). The initiating drivers directly trigger two distinct candidate pathways: the microbial/ecological pathway (green) and the microvascular/systemic pathway (orange). These pathways converge on tongue phenotypic signatures, including tongue color shift (L*, a*, b*, *Δ*E) and tongue-coating microbiota profiles. Confounding factors (e.g., image-acquisition and biological factors) may independently cause non-specific tongue changes. A predefined diagnostic comparison framework (NEC, sepsis without NEC, feeding intolerance/ileus without NEC, and stable preterm infants) is incorporated for specificity testing and is therefore presented as an analytical consideration rather than being linked mechanistically. Solid arrows denote better-supported relationships; colored dashed arrows denote hypothetical or analogical relationships; grey dash-dot arrows denote confounding non-causal interfering signals; blue workflow arrows indicate the sequential translational workflow (longitudinal analysis and predictive modelling → prospective validation with incremental utility assessment → potential clinical implementation and impact). NEC, necrotizing enterocolitis; L*, a*, b*, CIE Lab color space components; ΔE, overall color difference.

### 5.1 Phase I: discovery and exploratory association analysis

A prospective birth cohort of high-risk preterm infants (gestational age <32 weeks, birth weight <1,500 g) will be enrolled. We will adopt an infant-specific dynamic baseline estimated using longitudinal repeated-measures models to account for postnatal maturation, feeding transitions, and progressive microbial colonization. All tongue images will be acquired following the standardized protocols described in Section 4.1.

Tongue-image monitoring will be performed daily when clinically feasible, with frequency adjusted to clinical stability. Feeding status, infection biomarkers, and abdominal imaging findings will be recorded until NEC onset or discharge. Potential confounders will be prospectively collected and categorized as image-acquisition-related factors (illumination, color calibration, imaging distance, timing relative to feeding, and motion artifacts) and biological factors (gestational age, feeding mode, hemoglobin level, oxygenation, transfusion, vasopressor use, respiratory support, and systemic perfusion). All confounders will be adjusted for in statistical analyses.

Primary hypothesis testing focuses on intra-individual temporal changes relative to each infant's dynamic baseline. To distinguish NEC-specific tongue signatures from non-specific signals of general clinical deterioration, we adopt a four-group analytical framework: (i) NEC, (ii) sepsis without NEC, (iii) feeding intolerance/ileus without NEC, and (iv) stable preterm infants. Comparisons against sepsis without NEC and feeding intolerance/ileus without NEC constitute the primary assessment of diagnostic specificity; stable preterm infants are included for biological characterization and do not serve as the primary benchmark for specificity. All comparator groups will be enrolled from the outset where sample size permits. NEC is defined as Bell stage ≥ II, with standardized adjudication to differentiate it from spontaneous intestinal perforation, septic ileus, milk-protein-related disease, and congenital-heart-disease-associated intestinal hypoperfusion. Overlapping cases will be classified by primary diagnosis; sensitivity analyses using alternative schemes will assess robustness. Time-to-event analyses will be used to estimate the relevant temporal windows prospectively rather than applying prespecified thresholds.

### 5.2 Phase II: exploratory early-warning model development

We aim to develop a clinically applicable risk-assessment tool by integrating key quantitative tongue-image features derived from Phase I with well-established clinical risk factors (e.g., gestational age, birth weight, feeding status, infection biomarkers, abdominal findings, and antibiotic exposure). Machine-learning algorithms will be used to develop a predictive model, with preprocessing and feature selection embedded within cross-validation loops to reduce data leakage and overfitting. Dataset partitioning will be performed at the infant level to account for longitudinal repeated observations, with temporal splitting to exclude future information from prediction windows. NEC class imbalance will be handled during model training. Missing data will be addressed using appropriate statistical methods, and time-dependent covariates will be incorporated to account for the longitudinal monitoring design. Longitudinal modelling will account for repeated measurements within the same infant. Model performance will be evaluated via internal cross-validation; temporal and multicenter external validation will be conducted in future studies. Key metrics include sensitivity, specificity, AUROC, calibration, precision-recall AUC (PR-AUC), and decision-curve analysis. Performance will be assessed separately for NEC versus each comparator group to isolate NEC-specific tongue signatures from non-specific deterioration signals, with subgroup discrimination reported for adequately sized groups. The effective sample size depends on the number of NEC cases, not on the total number of tongue images. A comprehensive baseline clinical model, including all predictors available at the time of prediction (gestational age, birth weight, feeding status, antibiotic exposure, vital signs, hematologic and inflammatory parameters, respiratory and hemodynamic variables), will serve as the benchmark for assessing the incremental predictive value.

### 5.3 Phase III: mechanistic exploration and clinical translation

Building on the tongue-based predictive model developed in Phases I and II, this phase focuses on mechanistic exploration and stepwise clinical translation. At the mechanistic level, we will first characterize paired longitudinal multi-omics profiles of the tongue and stool (metagenomics and metabolomics) to map temporal associations and perform strain-level microbial source tracking. We will also measure circulating inflammatory and metabolic markers alongside tongue-surface microcirculation to quantify the dynamic link between time-dependent tongue color shifts and intestinal ischemic injury. This multi-omics approach draws on methodological precedent from cross-organ correlation analyses in non-NEC disease models (35). Targeted animal experiments will subsequently be designed to validate mechanistic signals emerging from the human cohort, rather than serving as a primary causal assay. At the clinical level, prospective multicenter trials will evaluate whether monitoring strategies guided by this candidate model can improve clinical outcomes, facilitating translation from research settings to the NICU bedside. These steps will be completed sequentially, and the model should not be used to guide therapeutic decisions until then.

## 6 Core testable scientific hypotheses

To move this framework from a conceptual construct toward a set of falsifiable research statements, three specific, quantifiable, exploratory hypotheses are proposed. H1 and H2 will be tested using data from the Phase I prospective cohort study, while H3 will be evaluated through model development and validation in Phase II (Table 1).

**Table 1:** Testable scientific hypotheses for tongue image-based NEC early warning.

Hypothesis	Core description	Quantitative metric	Exploratory temporal window/Parameter to be estimated
H1	Progressive intestinal mucosal ischemia may induce a detectable shift in tongue color from pale/pink toward bluish-purple, measurable at the bedside using standardized digital imaging.	Composite color metrics including L* (luminance), a*, b* (CIE Lab color space), and overall color difference ΔE	Temporal window to be prospectively estimated
H2	Gut dysbiosis preceding NEC may be reflected in compositional changes in the tongue-coating microbiome, detectable via non-invasive swab sampling.	α/β diversity, longitudinal trajectories, differential taxa, microbial load; functional pathways, strain-level source tracking, paired tongue–stool microbial relationships	Temporal window to be prospectively estimated
H3	A composite model combining tongue-based biomarkers and clinical risk factors may improve early prediction performance for NEC over clinical risk factors alone.	AUROC, calibration, PR-AUC, and decision-curve analysis	Incremental AUROC gain over a comprehensive full-variable clinical baseline model

Hypotheses are exploratory. Temporal windows and AUROC gain are parameters to be estimated prospectively and are not predefined thresholds. For H3, the primary parameter to be estimated is the incremental AUROC gain; calibration, PR-AUC, and decision-curve analysis will also be evaluated. CIE Lab, Commission Internationale de l'Eclairage Lab color space; AUROC, area under the receiver operating characteristic curve; PR-AUC, precision-recall AUC.

## 7 Challenges, limitations, and future perspectives

The application of tongue image analysis in early warning currently faces three major challenges. First, standardized acquisition protocols for preterm infants are not yet established. Image collection in preterm infants is frequently compromised by crying and oral secretions, necessitating specialized neonatal acquisition devices and adaptive image preprocessing algorithms. Second, the optimal timing and frequency of tongue-image monitoring for early NEC warning remain undefined. Third, the mapping between traditional diagnostic descriptors and modern quantitative indicators requires further refinement to establish a standardized system linking tongue phenotypes to specific pathological states.

A major limitation of the hypotheses presented in this Perspective is that direct clinical evidence on tongue image features in neonatal NEC is currently lacking, reflecting a broader research gap in the field. The correlations proposed herein are based on pathophysiological reasoning and indirect evidence from related fields (e.g., neonatal sepsis and intestinal microbiota research), including studies on the candidate oral–gut microbial relationship. As such, this work is intended to propose a testable research framework rather than to present validated conclusions. Nonetheless, this Perspective fills a critical gap by providing a systematic, actionable research roadmap for this emerging diagnostic modality, catalyzing interdisciplinary collaboration and prospective validation studies. Our research team will prioritize the three-phase framework to implement prospective cohort studies, generating direct clinical data and validating this conceptual framework.

Looking forward, if prospectively validated, this quantitative tongue image analysis framework may eventually be integrated into routine NICU care as a non-invasive monitoring modality. A portable, smart bedside device coupled with a cloud-based machine learning model could provide clinicians with a real-time, objective NEC risk score, primarily for prompting enhanced surveillance. Until prospectively validated, the proposed risk score should not be used to trigger bowel rest, anti-inflammatory therapy, or probiotics.

Clinical translation must proceed step-by-step through signal discovery, analytical validation, prospective clinical validation, evaluation of incremental utility, external validation, implementation studies, and clinical-impact trials. Reductions in NEC severity, surgical intervention rates, and mortality remain long-term aspirational objectives. This represents a potential step toward personalized, predictive medicine for our most vulnerable patients. Real-world deployment would also require cost-benefit evaluation and regulatory validation of neonatal-oriented hardware and algorithms before clinical adoption.

## Data Availability

The original contributions presented in the study are included in the article/Supplementary Material, further inquiries can be directed to the corresponding author.
